# IgA nephropathy: evolving treatment strategies

**DOI:** 10.1590/2175-8239-JBN-2026-0279en

**Published:** 2026-11-09

**Authors:** Gianna Mastroianni-Kirsztajn, Juliana Mansur Siliano, Eduardo de Paiva Luciano

**Affiliations:** 1 Universidade Federal de São Paulo, Departamento de Medicina, Disciplina de Nefrologia, São Paulo, SP, Brazil.

**Keywords:** Complement Activation, Corticosteroids, Glomerulonephritis, Immunoglobulin A, Glomerulonephritis, IgA, Renin-Angiotensin System, Endothelin Receptor Antagonists

## Abstract

IgA nephropathy (IgAN) is the most common primary glomerular disease and is characterized by marked clinical, laboratory, and histopathological heterogeneity, with a variable risk of progression to advanced stages of chronic kidney disease depending on several underlying factors. A better understanding of its pathogenesis, which involves at least a four-hit pathogenic sequence, has enabled the development of novel therapeutic strategies targeting different stages of disease development, including agents directed at specific molecular pathways. Supportive kidney-protective therapy is now well established as the cornerstone of IgAN management and involves lifestyle modifications such as a healthy diet, regular physical activity, weight control, smoking cessation, sodium restriction, optimal blood pressure control, and interventions to reduce cardiovascular risk. Pharmacological measures include renin-angiotensin-aldosterone system and sodium-glucose cotransporter 2 (SGLT2) inhibitors, as well as mineralocorticoid receptor and endothelin A receptor antagonists. Historically, immunosuppressive treatment relied primarily on systemic corticosteroids, with cyclophosphamide or azathioprine reserved for selected clinical situations. More recently, targeted-release budesonide has emerged as a therapeutic option, followed by the development of agents directed at specific molecular pathways, particularly complement inhibitors and therapies targeting BAFF and/or APRIL signaling, among others. Although this expanding therapeutic armamentarium provides new opportunities to improve patient outcomes, it also introduces uncertainty and complexity into clinical decision-making. Therefore, defining evidence-based parameters to guide the selection and combination of available therapies according to the distinct endotypes of IgAN has become increasingly important.

## INTRODUCTION

Immunoglobulin A nephropathy (IgAN) is the most common primary glomerulonephritis worldwide. More recently, it has become increasingly recognized as a highly heterogeneous disease, extending far beyond the classic presentation of isolated hematuria with a favorable long-term prognosis. In clinical practice, hematuria is more commonly accompanied by varying degrees of proteinuria, and approximately 20% to 40% of affected individuals eventually progress to kidney failure within 20 years after the disease onset^1^.

The current understanding of IgAN pathogenesis is based on a four-hit hypothesis, according to which disease development begins with (1) increased production of galactose-deficient IgA1 (Gd-IgA1) with aberrant glycosylation, followed by (2) the generation of anti-Gd-IgA1 autoantibodies, (3) the formation of circulating immune complexes containing Gd-IgA1, and finally (4) the mesangial deposition of these immune complexes, triggering glomerular inflammation and kidney injury^1^.

Growing evidence also indicates that the gut-associated lymphoid tissue (GALT) plays a pivotal role in the production of Gd-IgA1 through interactions between the intestinal microbiota and the mucosal immune system^1^.

Advances in the understanding of IgAN pathogenesis have led to the development of several novel therapeutic approaches targeting different stages of the disease cascade. Some of these therapies remain under clinical investigation, whereas others are already used in routine clinical practice.

The expanding therapeutic armamentarium has transformed the management of IgAN, but it has also introduced considerable uncertainty regarding the optimal treatment strategy for individual patients. One of the major unresolved questions is whether disease-specific pathogenic mechanisms and nonspecific consequences of nephron loss should be targeted simultaneously from the outset or whether a sequential, stepwise approach should be adopted, with additional therapies introduced only after the residual risk becomes apparent despite previous interventions^2^.

Proponents of early combination therapy argue that ongoing immune-mediated injury persists even after hemodynamic reduction of proteinuria, allowing glomerular damage to continue. Once a critical threshold of nephron loss has been exceeded, self-perpetuating hyperfiltration, progressive fibrosis, and microvascular rarefaction may ultimately limit the reversibility of kidney injury. Conversely, advocates of a sequential strategy emphasize the heterogeneous natural history of IgAN, the potential risks and costs associated with multiple therapies, and the importance of individualized treatment escalation based on dynamic risk assessment^2^.

Another important factor contributing to the rapid expansion of therapeutic options is the increasing pace at which new drugs are being approved by regulatory agencies and incorporated into clinical practice. Over the last decade, there has been a major shift in the required efficacy outcomes. Whereas earlier studies primarily relied on kidney function decline, kidney failure, or mortality as primary outcomes, proteinuria has now been validated and accepted as a surrogate endpoint for kidney disease progression and therapeutic response in IgAN, allowing treatment effects to be demonstrated much earlier during clinical development^3^.

Likewise, it is noteworthy that the target population eligible for treatment has changed considerably in recent years. Among several contributing factors, current international guidelines recommend a more liberal indication for kidney biopsy and more stringent proteinuria targets, aiming for levels <0.5 g/d, ideally <0.3 g/d, together with long-term preservation of kidney function^4^.

## DISCUSSION

Before addressing these issues in greater detail, it is worth briefly reviewing some of the major therapeutic strategies that have been available for the treatment of IgAN, as well as those currently under clinical development. Only selected therapies are discussed to illustrate the therapeutic landscape of IgAN over recent years, and the corresponding pivotal clinical trials are summarized in Chart 1. It remains uncertain which of these therapies will ultimately assume a leading role in clinical practice once their long-term efficacy and safety profiles have been fully established and compared.

**Chart 1:** Pivotal clinical trials that redefined the treatment of IgA nephropathy (2015–July 2026).

Study (year)	Target/therapeutic class	Study design and sample size	Population/race– ethnicity	Intervention and comparator (exact dose)	Follow-up/primary endpoint	Proteinuria outcome	eGFR outcome	Safety (key findings)	Regulatory status (as of July 11, 2026)	Clinical relevance and limitations
STOP-IgAN (2015) (Rauen et al.)^11^	Systemic immunosuppression	Multicenter, open-label, randomized trial; 337 patients entered the supportive-care run-in phase, with 162 subsequently randomized.	Germany; adults with IgA nephropathy and persistent proteinuria despite 6 months of optimized supportive care. Predominantly European population: detailed racial distribution was not emphasized in the primary publication.	eGFR ≥60 mL/min/1.73 m^2^: **glucocorticoid therapy** for 6 months (intravenous methylprednisolone 1 g/day for 3 consecutive days during months 1, 3, and 5, plus oral prednisolone 0.5 mg/kg every other day). eGFR 30–59 mL/min/1.73 m^2^: **cyclophosphamide** 1.5 mg/kg/day for 3 months, followed by **azathioprine** 1.5 mg/kg/day until month 36, with oral **prednisolone** initiated at 40 mg/day and gradually tapered. Comparator: intensive supportive care alone.	3 years. Co-primary endpoints: complete clinical remission and ≥15 mL/min/1.73 m^2^ decline in eGFR.	Complete remission: 17% with immunosuppression vs. 5% with supportive care (*p* = 0.01). Proteinuria was significantly reduced; however, this did not translate into long-term kidney protection.	≥15 mL/min/1.73 m^2^ eGFR decline: 26% vs. 28% (no significant difference). The chronic eGFR slope was similar between treatment groups.	Higher incidence of infections, >5-kg weight gain, glucose intolerance/diabetes, and one sepsis-related death in the immunosuppression group.	No disease-specific regulatory approval.	Established the importance of optimizing supportive care before initiating immunosuppressive therapy. Major limitations include the open-label design, relatively small sample size, different treatment regimens according to baseline eGFR, and the use of immunosuppressive strategies that are now considered excessively toxic.
STOP-IgAN – 10-year follow-up (2020) (Rauen et al.)^12^	Systemic immunosuppression	Observational follow-up of the randomized cohort; 149 patients with long-term follow-up data.	Same German cohort.	No additional protocol-defined intervention; comparison according to the original treatment allocation.	Median follow-up of approximately 7.4 years after randomization. Major kidney outcomes and all-cause mortality.	The initial antiproteinuric benefit was not sustained over long-term follow-up.	The composite kidney outcome occurred in 50% vs. 45% (immunosuppression vs. supportive care). No significant differences were observed in kidney failure, eGFR decline, or mortality.	No additional safety concerns beyond those reported in the original trial.	Not applicable.	Confirmed the absence of a long-term kidney benefit associated with the immunosuppressive strategy evaluated in STOP-IgAN.
TESTING – final results (2022) (Lv et al.)^13^	Systemic corticosteroids	International, randomized, double-blind trial; 503 patients (full-dose and reduced-dose regimens).	Sixty-seven centers across six countries. Approximately 95% of participants were Asian. Adults with IgA nephropathy, proteinuria ≥1 g/day and eGFR 20– 120 mL/min/1.73 m^2^.	Full-dose regimen: oral **methylprednisolone** 0.6–0.8 mg/kg/day (maximum 48 mg/day). Reduced-dose regimen: 0.4 mg/kg/day (maximum 32 mg/day) with a gradual taper over 6–9 months and *Pneumocystis* prophylaxis. Comparator: placebo.	Median follow-up of 4.2 years. Composite endpoint of: ≥40% decline in eGFR kidney failure kidney-related death	Greater proteinuria reduction was observed with methylprednisolone, particularly during the first year, although the effect gradually attenuated over time.	Primary kidney outcome: 28.8% vs. 43.1% HR 0.53 (95% CI 0.39–0.72). Kidney benefit was similar with both dosing regimens, whereas toxicity was lower with the reduced-dose protocol.	Serious adverse events: 10.9% vs. 2.8%, predominantly infections. Reduced-dose corticosteroids combined with *Pneumocystis* prophylaxis lowered, but did not eliminate, the risk of serious infections.	No disease-specific regulatory approval for IgA nephropathy.	Demonstrated meaningful kidney protfection, although at the expense of increased treatment-related toxicity. Its use is primarily applicable to carefully selected patients at high risk of progression. Generalizability remains limited by the predominance of Asian participants.
NEFIGAN (2017) (Fellström et al.)^14^	Targeted-release budesonide (Hit 1)	Phase 2b, randomized, double-blind trial; 150 patients.	European patients with IgA nephropathy and persistent proteinuria despite optimized RAAS blockade. Predominantly White population.	Targeted-release **budesonide**. 16 mg/day or 8 mg/day for 9 months versus placebo. All patients received optimized RAAS blockade.	9 months of treatment plus 3 months of follow-up. Primary endpoint: change in UPCR.	With the 16-mg dose, UPCR decreased by 27.3%, whereas placebo showed a 2.7% increase. The combined treatment groups demonstrated a statistically significant antiproteinuric effect.	eGFR remained stable in the budesonide groups and declined in the placebo group during treatment. The study was not powered for hard kidney endpoints.	Corticosteroid-related adverse events occurred but were generally milder than those observed with systemic corticosteroids. Hypertension and peripheral edema require clinical monitoring.	Provided the basis for the phase 3 NefIgArd trial.	First robust proof-of-concept demonstrating the efficacy of targeted mucosal therapy in IgA nephropathy. Limitations include the relatively short duration and reliance on a surrogate endpoint.
NefIgArd (2021– 2023) (Barrat et al.)^15^	Targeted-release budesonide (Hit 1)	Phase 3, randomized, double-blind trial; 364 patients.	Adults with IgA nephropathy enrolled across 20 countries, with UPCR ≥0.8 g/g or proteinuria ≥1 g/day, and eGFR 35–90 mL/min/1.73 m^2^. The population was predominantly White, with Asian patients underrepresented.	Targeted-release **budesonide** 16 mg/day for 9 months versus placebo, both added to optimized supportive care. Patients were subsequently followed for 15 months off-treatment.	Two years. Co-primary endpoints: UPCR at 9 months time-averaged eGFR over 2 years	Relative reduction in UPCR at 9 months of approximately 27% versus placebo. The antiproteinuric effect persisted partially after treatment discontinuation.	At 2 years, the time-averaged eGFR difference was approximately 5.0 mL/min/1.73 m^2^ in favor of budesonide. Kidney function declined significantly more slowly in the active-treatment group.	Higher rates of peripheral edema, hypertension, acne, and cushingoid features were observed. Systemic corticosteroid effects were reduced but not eliminated.	Full FDA approval for reducing kidney function loss in adults with primary IgA nephropathy at risk of progression (Tarpeyo). Also approved in other jurisdictions.	First disease-specific therapy to demonstrate an effect on eGFR after a finite treatment course. Limitations include exclusion of patients with eGFR below 35 mL/min/1.73 m^2^, limited Asian representation, and recurrence of proteinuria after treatment discontinuation.
PROTECT (2023; 2-year results) (Rovin et al.)^7^	Dual endothelin type A and angiotensin II receptor antagonism	Phase 3, randomized, double-blind, active-controlled trial; 404 patients.	Patients from 18 countries with IgA nephropathy, proteinuria ≥1 g/day, and eGFR ≥30 mL/min/1.73 m^2^. The study enrolled a global population that was predominantly White, with meaningful Asian representation.	**Sparsentan** 400 mg once daily versus irbesartan 300 mg once daily. Initial titration doses were 200 mg and 150 mg, respectively.	Week 36: change in UPCR. Up to week 110: eGFR slope.	At week 36, proteinuria decreased by 49.8% with sparsentan versus 15.1% with irbesartan, corresponding to a 41% relative between-group reduction. The effect was sustained through 2 years.	The chronic eGFR slope favored sparsentan by approximately 1.1 mL/min/1.73 m^2^ per year. The total eGFR slope did not meet statistical significance under the original analysis plan (*p* = 0.058).	Relevant adverse events included hypotension, edema, and liver enzyme abnormalities. Monitoring of liver function and pregnancy prevention is required. No disproportionate signal of heart failure was observed.	Full FDA approval (Filspari) to slow kidney function decline in adults with IgA nephropathy at risk of progression.	A highly potent antiproteinuric, nonimmunosuppressive option. It should not be combined with an ACE inhibitor or angiotensin receptor blocker. The eGFR findings were directionally favorable, although the prespecified total-slope endpoint was borderline.
ALIGN – Phase 3 (2026) (Heerspink et al.)^8^	Selective endothelin type A receptor antagonist	Phase 3, randomized, double-blind, placebo-controlled trial; 404 patients, including 340 in the main stratum and 64 in an exploratory SGLT2 inhibitor stratum.	Patients from 24 countries with IgA nephropathy, proteinuria ≥1 g/day, and eGFR ≥30 mL/min/1.73 m^2^, receiving maximally tolerated RAAS blockade. The population was global; detailed racial composition is provided in the study supplement.	**Atrasentan** 0.75 mg once daily versus placebo, added to supportive care.	Treatment for 132 weeks, followed by a 4-week off-treatment period, with final assessment at week 136.	A sustained reduction in proteinuria of approximately 38% relative to placebo.	Atrasentan slowed kidney function decline. The between-group difference in total eGFR slope was 1.4 mL/min/1.73 m^2^ per year in favor of atrasentan. At week 132, the between-group difference in eGFR change from baseline was 2.6 mL/min/1.73 m^2^.	Overall tolerability was favorable. Fluid retention was modest, and no new safety signals were identified.	FDA accelerated approval is undergoing conversion to traditional approval based on confirmatory kidney-function data.	Confirms long-term kidney benefit and supports atrasentan as a nonimmunosuppressive antiproteinuric therapy targeting the endothelin pathway. The prespecified week-136 eGFR endpoint narrowly missed statistical significance, although total eGFR slope and week-132 analyses favored atrasentan.
ENVISION (2024) (Mathur et al.)^21^	Monoclonal anti-APRIL antibody (Hit 1)	Phase 2, randomized, double-blind trial; 155 patients.	International study including adults with high-risk IgA nephropathy receiving supportive care. The population was racially diverse, although the study was not powered for subgroup analyses.	Subcutaneous **sibeprenlimab** administered every 4 weeks at doses of 2, 4, or 8 mg/kg versus placebo, for 12 months.	12 months. Primary endpoint: change in UPCR.	At 12 months, UPCR reductions were approximately: 47.2% with 2 mg/kg 58.8% with 4 mg/kg 62.0% with 8 mg/kg versus approximately 20% with placebo, demonstrating a dose-response relationship.	eGFR remained more stable in the active-treatment groups. However, the phase 2 trial was not powered to assess definitive kidney outcomes.	Overall adverse-event rates were similar across groups. The most relevant events were respiratory infections and injection-site reactions. Serum IgA levels decreased without broad IgG depletion.	No regulatory approval based solely on ENVISION.	Validated APRIL as a causal therapeutic target and supported progression to phase 3 development. Main limitations were the small sample size and relatively short follow-up.
VISIONARY – Interim analysis (2025) (Perkovic et al.)^22^	Monoclonal anti-APRIL antibody (Hit 1)	Phase 3, multicenter, randomized, double-blind trial; approximately 510 randomized participants, with a prespecified interim proteinuria analysis.	Adults with biopsy-confirmed IgA nephropathy at risk of disease progression enrolled in a global study. Detailed racial distribution is reported in the supplementary appendix.	Subcutaneous sibeprenlimab 400 mg every 4 weeks versus placebo for 100 weeks.	Proteinuria (UPCR) at 9 months. Follow-up for eGFR outcomes is ongoing through 24 months.	Adjusted relative reduction in UPCR versus placebo of approximately 51.2% at 9 months.	The confirmatory eGFR endpoint remains under evaluation in the ongoing phase 3 study.	Safety profile comparable to placebo. Most adverse events consisted of mild-to-moderate infections and injection-site reactions.	FDA accelerated approval (November 2025; Voyxact^®^) for reduction of proteinuria in adults with IgA nephropathy. Continued approval is contingent upon confirmation of long-term kidney benefit.	First anti-APRIL therapy to receive regulatory approval. Demonstrated a marked antiproteinuric effect with a strong disease-modifying rationale; however, confirmation of long-term kidney protection is still awaited.
ORIGIN- 3 – Interim analysis (2025) (Lafayette et al.)^24^	Dual BAFF/APRIL blockade (TACI-Fc) (Hit 1)	Phase 3, randomized, double-blind, placebo-controlled trial; 431 patients.	Global study including patients with persistent proteinuria and risk of progression despite optimized supportive care. Complete racial distribution is available in the original publication and supplementary appendix.	Self-administered subcutaneous **atacicept** 150 mg once weekly versus placebo. Change in UPCR at week 36. Follow-up for kidney outcomes is ongoing.	Adjusted relative reduction versus placebo of 41.8%, corresponding to an approximately 46% reduction from baseline with atacicept.	Definitive kidney outcome data are not yet available from the published interim analysis.	Treatment was associated with reductions in serum IgA, IgG, and IgM concentrations. Overall infection rates were comparable between groups; however, monitoring of immunoglobulin levels and vaccination status is recommended.	FDA accelerated approval (July 7, 2026; Trutakna®) for reduction of proteinuria. Continued approval depends on confirmation of kidney-function benefit.	First dual BAFF/APRIL inhibitor approved for IgA nephropathy. Produced robust improvements in proteinuria and disease-related biomarkers; however, treatment cost, immunoglobulin reduction, and the absence of definitive kidney outcome data remain important limitations.
TELIGAN – Interim analysis (2026) (Lv et al.)^25^	Dual BAFF/APRIL blockade (TACI-Fc) (Hit 1)	Phase 3, randomized, double-blind, placebo-controlled trial conducted predominantly in China.	Chinese adults with high-risk IgA nephropathy. Generalizability to other ethnic populations remains limited.	Subcutaneous **telitacicept** 240 mg once weekly versus placebo, in addition to optimized supportive therapy.	Change in UPCR at 39 weeks. Follow-up for kidney-function outcomes is ongoing.	Significantly greater reduction in UPCR compared with placebo at week 39. The published treatment effect was approximately 40–50% relative reduction, according to the interim analysis.	No definitive evidence of kidney-function preservation is yet available.	Reductions in immunoglobulin levels and infections require monitoring. Overall safety profile was acceptable in the interim analysis.	Approved in China for selected autoimmune diseases. The regulatory status for IgA nephropathy varies across jurisdictions and no FDA approval had been granted as of July 11, 2026.	Further supports the therapeutic potential of BAFF/APRIL inhibition. However, the predominantly Chinese population and the absence of definitive kidney outcome data limit extrapolation to broader populations.
APPLAUSE-IgAN - Interim and 24-month results (2025–2026) (Barratt et al.)^19^	Oral factor B inhibitor – alternative complement pathway (Hit 4)	Phase 3, randomized, double-blind, placebo-controlled trial. 518 patients in the primary study population, with an additional cohort including patients with advanced chronic lesions.	International study including patients with IgA nephropathy, proteinuria ≥1 g/day, and eGFR ≥30 mL/min/1.73 m^2^.	Oral **iptacopan** 200 mg twice daily versus placebo, both added to optimized supportive therapy.	Proteinuria (UPCR) at 9 months. Total eGFR slope and safety evaluated over 24 months.	Adjusted relative reduction in UPCR of 38.3% versus placebo at month 9.	Final 24-month results demonstrated a significantly slower decline in eGFR with iptacopan. The total eGFR slope consistently favored active treatment (see the final publication for the exact estimate and confidence intervals).	Overall infection rates were similar between treatment groups. Because of the mechanism of complement inhibition, vaccination and patient education regarding encapsulated bacterial infections are recommended. No clinically relevant signal for hemolysis was observed.	FDA accelerated approval (Fabhalta®) for reduction of proteinuria in adults with IgA nephropathy. The 24-month results provide confirmatory evidence supporting long-term kidney benefit.	First complement-targeted therapy to demonstrate phase 3 evidence of both substantial proteinuria reduction and preservation of kidney function. Treatment cost, vaccination requirements, and future biomarker-guided patient selection remain important practical considerations.

Abbreviations – eGFR, estimated glomerular filtration rate; UPCR, urinary protein-to-creatinine ratio; RAAS, renin-angiotensin-aldosterone system; APRIL, a proliferation-inducing ligand; BAFF, B-cell activating factor; TACI, transmembrane activator and CAML interactor.

Notes – This chart summarizes pivotal clinical trials evaluating systemic immunosuppression and targeted therapies directed against pathogenic IgA production, the endothelin pathway, and the complement system. Data are presented according to the primary publications. Interim phase 3 analyses should not be interpreted as definitive evidence of kidney-function preservation until confirmatory eGFR outcome data become available. Critical note: Accelerated regulatory approvals based primarily on reductions in proteinuria remain contingent upon confirmation of long-term clinical benefit on kidney function.

### Treatment strategies

#### Supportive kidney-protective therapy

Over the past decade, it has become increasingly evident that a substantial proportion of IgAN progression results from self-perpetuating intrarenal mechanisms of injury that persist independently of the initial immune trigger. Activation of pathways involved in intraglomerular hypertension, podocyte dysfunction, tubulointerstitial inflammation, and progressive fibrosis is now recognized as a major therapeutic target.

Accordingly, individualized and optimized supportive kidney-protective therapy is currently considered the foundation of IgAN management. Depending on the patient’s clinical presentation and the therapeutic strategy adopted, these measures may either precede or be initiated concomitantly with disease-specific immunomodulatory therapy, although the optimal timing remains a matter of ongoing debate^4^.

Supportive care includes lifestyle interventions such as adherence to a healthy diet, regular physical activity, weight control, smoking cessation, sodium restriction, optimal blood pressure control, and measures aimed at reducing cardiovascular risk.

The pharmacological cornerstone of supportive therapy currently comprises (1) renin-angiotensin-aldosterone system (RAAS) inhibitors, (2) sodium-glucose cotransporter 2 (SGLT2) inhibitors, (3) mineralocorticoid receptor, and (4) endothelin A receptor antagonists. Some of these therapies, particularly RAAS blockade and SGLT2 inhibitors, have demonstrated clear benefits not only in chronic kidney disease and proteinuric glomerular disorders in general but also specifically in IgAN^5,6^. Consequently, they have become integral components of routine clinical practice worldwide.

Among emerging kidney-protective therapies, endothelin receptor antagonists represent one of the most promising advances. Endothelin-1 contributes to podocyte injury, increased glomerular permeability, intrarenal vasoconstriction, and progression of kidney fibrosis. In the PROTECT trial, sparsentan, a dual endothelin type A (ETA) and angiotensin II type 1 (AT1) receptor antagonist, produced a sustained reduction in proteinuria accompanied by a slower decline in kidney function compared with irbesartan, establishing itself as an important therapeutic option for patients with IgAN at high risk of progression^7^.

More recently, the ALIGN trial demonstrated that selective ETA receptor blockade with atrasentan significantly reduced proteinuria when added to optimized supportive therapy, further confirming the endothelin pathway as an independent therapeutic target beyond immune modulation^8^.

Another strategy that has gained increasing attention is nonsteroidal mineralocorticoid receptor antagonism. Although finerenone was initially developed for diabetic kidney disease, the FIND-CKD trial demonstrated that it also reduced albuminuria and proteinuria while slowing the decline in estimated glomerular filtration rate (eGFR) in patients with non-diabetic chronic kidney disease, including a large population with glomerular diseases and a substantial subgroup of patients with IgAN^9^.

The following sections summarize additional therapeutic approaches, including immunomodulatory agents currently used in clinical practice, as well as selected clinical trials that illustrate the evolution of IgAN treatment over the past several decades.

#### Corticosteroids

Although earlier studies demonstrated meaningful clinical benefits with systemic corticosteroid therapy in IgAN, the use of corticosteroids has become increasingly controversial over the past decade. This shift has been driven largely by more recent clinical trials highlighting both the limitations and the adverse effects associated with systemic corticosteroid therapy.

Nevertheless, it is important to emphasize that systemic corticosteroids remain unquestionably indicated in selected clinical scenarios, particularly in patients presenting with rapidly progressive glomerulonephritis or nephrotic syndrome^10^ at least until more effective and safer therapeutic alternatives become available.

The publication of the STOP-IgAN (Supportive Versus Immunosuppressive Therapy for the Treatment of Progressive IgA Nephropathy) trial represented a major turning point in the management of IgAN. Following a period of rigorous optimization of supportive care, the addition of systemic immunosuppression—consisting of corticosteroids for all patients, with cyclophosphamide followed by azathioprine in those with an estimated glomerular filtration rate (eGFR) between 30 and 59 mL/min/1.73 m^2^—failed to provide sustained long-term preservation of kidney function, despite achieving higher rates of clinical remission and greater reductions in proteinuria in a subset of patients. In addition to the lack of a consistent benefit on long-term eGFR decline, the increased incidence of adverse events reinforced the need for a more careful selection of candidates for systemic immunosuppressive therapy^11,12^.

Several years later, the TESTING trial provided a renewed perspective by demonstrating that oral methylprednisolone significantly reduced the risk of kidney disease progression in IgAN. However, the first phase of the study (TESTING-1), which evaluated high-dose methylprednisolone (0.8 mg/kg/day), was terminated prematurely because of an excess of serious infections, highlighting the need to balance therapeutic efficacy against treatment-related toxicity. A subsequent protocol (TESTING-2) employed a lower dose of methylprednisolone (0.4 mg/kg/day) together with *Pneumocystis jirovecii* prophylaxis, preserving the renoprotective benefit while substantially improving the safety profile. These findings consolidated systemic corticosteroids as a valuable therapeutic option for carefully selected patients at high risk of progression, although they clearly do not represent a universal treatment strategy for all individuals with IgAN^13^.

An important opportunity to reduce corticosteroid-related toxicity emerged with the development of targeted-release budesonide, which introduced a fundamentally different therapeutic concept in IgAN. This formulation was specifically designed to deliver the drug to the distal ileum, where gut-associated lymphoid tissue (GALT)—one of the principal sites of pathogenic Gd-IgA1 production—is concentrated. By modulating mucosal immune activity at its presumed source, targeted-release budesonide aims to reduce disease activity while minimizing systemic corticosteroid exposure.

The NEFIGAN and, subsequently, the NefIgArd trials demonstrated significant and sustained reductions in proteinuria accompanied by a slower decline in kidney function among patients with IgAN at high risk of progression, establishing targeted-release budesonide as the first disease-specific therapy approved for IgAN^14,15^. Beyond its clinical efficacy, these studies introduced a concept that has since become central to the development of novel therapies for IgAN: the possibility of selectively targeting key pathogenic mechanisms rather than relying on nonspecific systemic immunosuppression.

#### Cyclophosphamide, mycophenolate, and other immunomodulatory agents

In addition to corticosteroids, cyclophosphamide and azathioprine have historically been used in selected patients with proteinuric IgAN, generally in combination with corticosteroid therapy. However, their use has become increasingly restricted to specific clinical settings, particularly rapidly progressive disease.

Mycophenolate mofetil (MMF) is currently recommended by international guidelines as a potential alternative to systemic corticosteroids; however, this recommendation is limited to Chinese patients. In this population, randomized clinical trials with long-term follow-up have demonstrated that MMF preserves kidney function while reducing the need for corticosteroid exposure. To date, these benefits have not been consistently reproduced in predominantly Caucasian populations^16^.

Similarly, hydroxychloroquine has demonstrated favorable effects in Chinese patients with IgAN, including significant reductions in proteinuria and a favorable safety profile. However, comparable evidence is lacking in non-Chinese populations, and its routine use outside this ethnic group cannot currently be recommended^17,18^.

#### Complement inhibitors

A growing body of experimental and clinical evidence has established activation of the alternative and lectin complement pathways as a key driver of disease progression in IgAN. Glomerular deposition of complement components—including C3, properdin, C4d, and other complement proteins—is frequently associated with greater disease activity and a worse renal prognosis, providing a strong biological rationale for the development of therapies targeting specific components of the complement cascade.

Among these strategies, inhibition of complement factor B, a key component of the alternative pathway, has generated the most robust clinical evidence to date. The APPLAUSE-IgAN trial demonstrated that iptacopan, an orally administered selective factor B inhibitor, produced a significant and sustained reduction in proteinuria, accompanied by a slower decline in kidney function and a lower risk of composite renal outcomes during follow-up. These findings established alternative pathway inhibition as an effective therapeutic strategy in IgAN and ultimately led to the regulatory approval of iptacopan for patients at risk of disease progression^19^.

Several additional complement-targeted therapies are currently under clinical investigation, with encouraging preliminary results demonstrating reductions in proteinuria. These agents target different components of the complement cascade, including C3 (e.g., pegcetacoplan), C5 (e.g., ravulizumab), the C5a receptor, factor D of the alternative pathway, and MASP-2, a key mediator of the lectin pathway, among others^20^.

#### Therapies targeting BAFF and/or APRIL signaling

The first generation of systemic therapies specifically designed to interfere with the production of Gd-IgA1—the initiating event in IgAN—aims to intervene early in the pathogenic cascade by reducing the formation of immune complexes before glomerular deposition occurs. Among the molecular targets currently under investigation, modulation of A Proliferation-Inducing Ligand (APRIL) and B-cell Activating Factor (BAFF) has emerged as the most advanced therapeutic strategy.

APRIL and BAFF play central roles in B-cell survival, maturation, differentiation, and plasma cell function, thereby regulating immunoglobulin production, particularly IgA. Increased circulating levels of these cytokines in patients with IgAN are correlated with higher serum Gd-IgA1 levels, greater proteinuria, and an increased risk of disease progression.

The first successful therapeutic approach focused on selective APRIL inhibition. In the ENVISION trial, sibeprenlimab significantly reduced proteinuria while producing sustained reductions in circulating IgA and Gd-IgA1 concentrations, providing proof of concept that selective APRIL blockade could modify biomarkers directly linked to disease pathogenesis. These findings were subsequently confirmed in the phase 3 VISIONARY trial, which demonstrated similar efficacy in a larger patient population and established sibeprenlimab as the first anti-APRIL therapy approved for patients with IgAN at risk of progression^21,22^

A complementary strategy has sought to achieve broader immunomodulation through the simultaneous inhibition of both APRIL and BAFF. Atacicept, a recombinant fusion protein derived from the transmembrane activator and CAML interactor (TACI) receptor, produced consistent reductions in proteinuria together with marked decreases in circulating IgA and Gd-IgA1 levels in the ORIGIN trial. These findings were subsequently confirmed in the phase 3 ORIGIN-3 study^23,24^. In July 2026, atacicept became the first dual APRIL/BAFF inhibitor to receive FDA approval for reducing proteinuria in patients with IgAN.

Telitacicept, which shares a similar mechanism of action, has likewise demonstrated substantial reductions in proteinuria in phase 3 clinical trials, further supporting the therapeutic potential of dual APRIL/BAFF blockade. Nevertheless, additional evidence regarding long-term preservation of kidney function and safety is still required before its role in routine clinical practice can be fully established^25^.

Importantly, direct comparisons among the currently available APRIL- and BAFF-targeted therapies should be avoided, as the respective clinical trials differed substantially with regard to inclusion criteria, background supportive therapy, duration of follow-up, and primary efficacy endpoints. Consequently, there is currently no evidence supporting the superiority of one agent over another.

For the first time, it has become possible to selectively modulate the systemic production of pathogenic IgA through mechanism-based interventions, moving IgAN treatment beyond nonspecific immunosuppression and toward precision medicine guided by disease pathophysiology.

#### Anti-CD38 therapies and others

In addition to the therapeutic approaches described above, anti-CD38 monoclonal antibodies are currently under clinical investigation for the treatment of IgAN. These agents selectively deplete CD38-positive plasma cells, which are believed to be major producers of both Gd-IgA1 and anti-Gd-IgA1 autoantibodies, with encouraging initial results suggesting that plasma cell-directed therapy may represent a disease-modifying strategy^26^. Podocyte inflammation inhibitors (anti-suPAR) and other agents are also under investigation^27^.

## CONCLUSION

There have undoubtedly been major advances in the therapeutic management of IgAN, as well as other glomerular diseases, after decades of limited therapeutic innovation. A better understanding of the pathogenic mechanisms underlying IgAN has contributed to the development of drugs targeting specific molecular pathways involved in disease pathogenesis. These therapies offer a new opportunity to delay or potentially halt kidney injury in patients with IgAN.

Although the availability of multiple therapeutic options is encouraging, selecting the most appropriate treatment strategy has become increasingly challenging. As an example, it is worth considering the current situation in a country such as Brazil. First, treatment should always be individualized according to the clinical, laboratory, and histopathological characteristics of IgAN, as well as patient-specific factors, including comorbidities and genetic background.

Most of the kidney-protective therapies discussed in this review are already available in Brazil and should be used in combination whenever appropriate, aiming to achieve the greatest possible reduction in proteinuria while preserving kidney function. Once available, endothelin receptor antagonists will certainly represent an important additional therapeutic option to be used in combination, particularly with RAAS blockade, as well as with other kidney-protective therapies. Potential adverse effects should be carefully considered when selecting these combinations. At present, RAAS blockade remains the cornerstone of supportive therapy, with additional agents being incorporated as needed. The decision to initiate immunosuppressive therapy also depends on the patient’s clinical presentation and should be based on identifying those individuals most likely to benefit from immune modulation. Currently, systemic corticosteroids remain the mainstay of treatment for patients presenting with nephrotic syndrome or rapidly progressive glomerulonephritis, with the option of combining them with other immunosuppressive agents when indicated. The greatest challenge lies in managing the wide spectrum of non-nephrotic presentations, with or without impaired kidney function, for which the clinician must determine the extent to which modulation of the immune system is appropriate and for how long. In these situations, systemic corticosteroids remain the only immunosuppressive agents routinely available, with current recommendations favoring lower-dose regimens.

It is important to emphasize that reliable histopathological and laboratory biomarkers capable of clearly guiding therapeutic decisions are still lacking. Likewise, no single instrument is currently available to accurately define risk categories in IgAN and consistently support individualized treatment decisions^28^. Persistent proteinuria despite optimized supportive therapy, more pronounced hematuria, and limited chronic changes on kidney biopsy are general indicators of ongoing disease activity and potential responsiveness to treatment. When approved and available, targeted immunomodulatory therapies may be considered in these patients after careful assessment of the risk-benefit balance on an individual basis.

It is of note that, given the concern regarding disease progression, patients with proteinuric IgAN are increasingly being encouraged to participate in clinical trials conducted at centers actively involved in research on therapeutic strategies^10^.

Although some of the novel therapies discussed here have already been approved for IgAN, their costs remain high, limiting patient access to them, which is an additional factor to be considered when prescribing such agents. Therefore, a critical approach should be maintained until their indications across the different clinical presentations and stages of progression of IgAN are more clearly established.

Finally, only a few therapeutic agents have been discussed here to illustrate the current landscape of treatment options for IgAN. Further studies are needed to determine the most appropriate therapeutic combinations. It is already becoming evident, however, that the future of IgAN treatment will not consist of a single therapeutic approach for a “single disease,” but rather of individualized treatment combinations tailored to the different endotypes of this glomerular disorder and to the specific needs of each patient, including their well-being and quality of life.

## Data Availability

No new data were generated or analyzed in this study.
