# Supplementary material for: IgA nephropathy: evolving treatment strategies
Source: J Bras Nefrol. 2026 Nov 9;49(1):e20260279. doi: 10.1590/2175-8239-JBN-2026-0279en (PMC13747299; doi:10.1590/2175-8239-JBN-2026-0279en)
Supplement: Translation

# Translation: Nefropatia por IgA: estratégias de tratamento em evolução

**DOI:** 10.1590/2175-8239-JBN-2026-0279pt

**Authors:** Gianna Mastroianni-Kirsztajn, Juliana Mansur Siliano, Eduardo de Paiva Luciano

**Affiliations:** 1 Universidade Federal de São Paulo, Departamento de Medicina, Disciplina de Nefrologia, São Paulo, SP, Brasil.

**Keywords:** Ativação do Complemento, Corticosteroides, Glomerulonefrite, Imunoglobulina A, Glomerulonefrite por IgA, Sistema Renina-Angiotensina, Antagonistas dos Receptores de Endotelina

## Abstract

A nefropatia por IgA é considerada a mais frequente entre as doenças glomerulares, tem apresentações clínico-laboratoriais e histopatológicas bastante diversas e potencial de progressão para estágios avançados de DRC conforme diversas características subjacentes. O maior conhecimento da sua patogênese, que envolve pelo menos uma sequência de 4 fatores desencadeantes principais, possibilitou o desenvolvimento de alternativas terapêuticas, que podem atuar em diferentes etapas de sua gênese, inclusive fármacos direcionados a alvos específicos ao longo desse processo. Já está bem definido que as medidas ditas renoprotetoras são essenciais para o tratamento de suporte, envolvendo modificação no estilo de vida (dieta saudável, exercícios físicos, controle de peso, cessação do tabagismo, restrição de sódio, controle da pressão arterial e medidas para redução do risco cardiovascular) e uso de fármacos, entre os quais devem ser mencionados: inibidores do SRAA e do SGLT2, antagonistas do receptor de mineralocorticoide e do receptor de endotelina A. Os recursos imunossupressores concentraram-se no passado, sobretudo, no uso de corticosteroide sistêmico, aliado à ciclofosfamida ou à azatioprina em situações especiais. Mais recentemente, surgiu a budesonida de liberação entérica, seguindo-se o desenvolvimento de fármacos direcionados a vias moleculares específicas, entre os quais se destacam os inibidores do complemento e aqueles direcionados à sinalização BAFF e/ou APRIL, entre outros. Essa diversidade de medicamentos gera oportunidades, mas também dúvidas e desafios por ocasião da tomada de decisão, exigindo definição de parâmetros que guiem a seleção das opções terapêuticas e suas combinações em prol do melhor resultado para o paciente, diante dos diferentes endótipos dessa glomerulopatia.

## INTRODUÇÃO

A nefropatia por imunoglobulina A (NIgA) é vista como a glomerulonefrite primária mais comum em todo o mundo. Mais recentemente, tem-se reconhecido que sua apresentação clínica é bastante heterogênea, indo além dos quadros de hematúria isolada com boa evolução ao longo dos anos. De fato, mais comumente, a proteinúria de diversos graus associa-se à hematúria, e 20% a 40% dos pacientes chegam a desenvolver doença renal terminal em até 20 anos após o início da doença^1^.

Entende-se, hoje, a patogênese da NIgA como um processo sequencial que envolve quatro fatores desencadeantes principais. A hipótese dos quatro “gatilhos” postula que: (1) ocorre inicialmente uma produção aumentada de uma IgA1 deficiente em galactose (Gd-IgA1) e glicosilada de forma aberrante, (2) seguida pela formação de autoanticorpos anti-Gd-IgA1, (3) com posterior formação de complexos imunes contendo IgA1, os quais (4) se depositam no mesângio glomerular, levando à inflamação e à lesão^1^. Há também indícios de que o tecido linfoide associado ao intestino desempenha um papel patogênico fundamental na produção da Gd-IgA1, por meio das interações entre a microbiota e a mucosa intestinal^1^.

O maior conhecimento da patogênese da NIgA possibilitou, nos últimos anos, o desenvolvimento de alternativas terapêuticas, que podem atuar em diferentes etapas de sua gênese, inclusive fármacos direcionados a alvos específicos ao longo desse processo. Algumas delas ainda estão em avaliação (pesquisa clínica) e outras já se encontram disponíveis para uso na prática diária.

Essa diversidade de fármacos e abordagens terapêuticas tem causado uma reviravolta no meio médico, assim como gerado muitas dúvidas no momento de decidir qual a melhor conduta diante do paciente com NIgA.

Um desses desafios consiste em definir se o mais correto é intervir simultaneamente nos mecanismos patogênicos específicos da NIgA e nas consequências inespecíficas da perda de néfrons, ou seguir uma abordagem sequencial e gradual, na qual as terapias são adicionadas somente ao se tornar evidente o risco residual após a adição de cada conduta terapêutica^2^.

Para alguns pesquisadores, a proposta tradicional de maximizar primeiramente os cuidados de suporte visando à proteção renal, antes de iniciar imunossupressores ou outros agentes direcionados a alvos específicos da doença glomerular, acarreta um atraso prejudicial. Eles ressaltam que a NIgA mantém uma atividade imune contínua e a lesão glomerular progride, mesmo quando a proteinúria é hemodinamicamente reduzida. Consideram que, uma vez ultrapassado um limiar crítico de perda de néfrons, a hiperfiltração que se autoperpetua, a fibrose e a rarefação microvascular limitam a reversibilidade. Já os defensores de uma estratégia “sequencial” destacam a heterogeneidade do curso da doença, os riscos e custos do uso de muitos medicamentos e a necessidade de escalonamento individualizado com base na avaliação dinâmica do risco^2^.

Outro aspecto que precisa ser abordado e que contribui para a oferta dessa diversidade de alternativas terapêuticas é o aumento da velocidade com que os novos fármacos estão sendo aprovados por agências reguladoras e disponibilizados para uso na prática clínica. Houve uma mudança na última década em relação aos desfechos exigidos para comprovar a eficácia dos fármacos nos ensaios clínicos, que antes se baseavam sobretudo em avaliação da redução da taxa de filtração glomerular (TFG), desenvolvimento de insuficiência renal e/ou óbito. Nos dias de hoje, a proteinúria é reconhecida como um marcador substituto da progressão da doença renal e da resposta a intervenções terapêuticas, podendo ser avaliada mais precocemente no curso dos estudos^3^.

Vale ressaltar ainda que a população-alvo dos tratamentos propostos para NIgA vem mudando nos últimos tempos. Entre outros possíveis motivos para isso, é notório que diretrizes internacionais atualmente incentivam uma política mais liberal para biópsia renal e sugerem um controle mais rigoroso da proteinúria, com metas <0,5 g/dia, idealmente <0,3 g/dia, e manutenção de TFG estável^4^.

## DISCUSSÃO

Antes de discutirmos em mais detalhes essas questões, é importante recordar brevemente algumas das principais opções de tratamento que estiveram e estão à disposição dos pacientes com NIgA e, em especial, as que se encontram em desenvolvimento. Apenas algumas delas serão mencionadas a seguir para ilustrar a evolução nessa área, e os respectivos estudos serão apresentados de forma esquemática no Quadro 1. Não é possível dizer neste momento quais delas assumirão posições de destaque na prática clínica, quando os seus perfis de eficácia e segurança forem comparados.

**Quadro 1.** Ensaios clínicos que redefiniram o tratamento da nefropatia por IgA (2015–julho de 2026).

Estudo (ano)	Alvo/classe terapêutica	Desenho e N	População/ raça-etnia	Intervenção e comparador (dose exata)	Seguimento/ desfecho primário	Resultado de proteinúria	Resultado de TFGe	Segurança (achados mais relevantes)	Status regulatório até 11/07/2026	Impacto real e limitações
STOP-IgAN (2015) (Rauen et al.)^11^	Imunossupressão sistêmica	Multicêntrico, aberto, randomizado; 337 na fase de suporte e 162 randomizados.	Alemanha; adultos com IgAN e proteinúria persistente após 6 meses de suporte otimizado. Predominantemente europeus; distribuição racial detalhada não foi destaque na publicação principal.	TFGe ≥60: **glicocorticoide** por 6 meses (metilprednisolona IV 1 g/d por 3 dias nos meses 1, 3 e 5 + prednisolona oral 0,5 mg/kg em dias alternados). TFGe 30–59: **ciclofosfamida** 1,5 mg/kg/d por 3 meses, seguida de **azatioprina** 1,5 mg/kg/d até o mês 36, com **prednisolona** oral inicial 40 mg/d e desmame. Comparador: suporte intensivo isolado.	3 anos. Coprimários: remissão clínica completa e queda da TFGe ≥15 mL/ min/1,73 m^2^.	Remissão completa: 17% com imunossupressão vs. 5% com suporte (*p* = 0,01); redução de proteinúria sem tradução em proteção funcional.	Queda da TFGe ≥15: 26% vs. 28% (sem diferença); inclinação da TFGe semelhante.	Mais infecções, ganho >5 kg, intolerância à glicose/diabetes; 1 óbito por sepse no grupo imunossupressão.	Sem indicação regulatória específica.	Estabeleceu a necessidade de otimizar suporte antes de imunossuprimir. Limitado pelo desenho aberto, pequeno *N*, regimes distintos conforme TFGe e uso de terapia hoje considerada excessivamente tóxica.
STOP-IgAN – seguimento de 10 anos (2020) (Rauen et al.)^12^	Imunossupressão sistêmica	Seguimento observacional da coorte randomizada; 149 pacientes com dados de longo prazo.	Mesma coorte alemã.	Sem nova intervenção protocolar; comparação segundo alocação original.	Mediana ~7,4 anos após randomização; desfechos renais maiores e óbito.	O benefício antiproteinúrico inicial não se traduziu em vantagem sustentada.	Desfecho composto em 50% vs. 45% (imunossupressão vs. suporte); nenhuma diferença em insuficiência renal, queda de TFGe ou óbito.	Não acrescenta segurança além do estudo original.	Não aplicável.	Confirmou ausência de benefício renal tardio da estratégia imunossupressora do STOP-IgAN.
TESTING – resultados finais (2022) (Lv et al.)^13^	Corticosteroide sistêmico	Internacional, duplo-cego, randomizado; 503 pacientes (regimes de dose plena e reduzida).	67 centros em 6 países; aproximadamente 95% asiáticos proteinúria ≥1 g/d e TFGe 20–120.	Dose plena: 0,6–0,8 mg/kg/d (máx. 48 mg). Dose reduzida: 0,4 mg/kg/d (máx. 32 mg), com desmame por 6–9 meses e profilaxia para *Pneumocystis*. Placebo.	Mediana 4,2 anos. Composto de queda ≥40% da TFGe, insuficiência renal ou morte renal.	Redução de proteinúria maior no grupo metilprednisolona, mais pronunciada no primeiro ano; efeito atenuado posteriormente.	28,8% vs. 43,1%; HR 0,53 (IC95% 0,39–0,72). Benefício semelhante nos regimes, com menor toxicidade na dose reduzida.	Eventos adversos graves: 10,9% vs. 2,8%; sobretudo infecções. Risco menor após dose reduzida + profilaxia, mas não eliminado.	Sem indicação específica aprovada para IgAN.	Demonstra eficácia renal, mas com custo de segurança. Aplicável sobretudo a pacientes de alto risco cuidadosamente selecionados; generalização limitada pela predominância asiática.
NEFIGAN (2017) (Fellström et al.)^14^	Budesonida de liberação direcionada ao íleo (“Gatilho” 1)	Fase 2b, duplo-cego, randomizado; 150 pacientes.	Europa; IgAN com proteinúria persistente apesar de bloqueio do SRAA. População majoritariamente branca.	**Budesonida** de liberação direcionada 16 mg/d ou 8 mg/d por 9 meses vs. placebo; todos com SRAA otimizado.	9 meses + 3 meses de observação. Primário: mudança no UPCR.	16 mg: redução de 27,3% no UPCR vs. aumento de 2,7% com placebo; efeito combinado das doses significativo.	TFGe permaneceu estável com budesonida e caiu no placebo durante o período de tratamento; estudo não dimensionado para desfecho renal duro.	Eventos relacionados a corticosteroide ocorreram, mas em geral menos intensos que com esteroide sistêmico; hipertensão e edema exigem monitorização.	Base para desenvolvimento do NefIgArd.	Primeira prova robusta de conceito de terapia mucosa direcionada; curta duração e *endpoint* substituto.
NefIgArd (2021-2023) (Barrat et al.)^15^	Budesonida de liberação direcionada ao íleo (“Gatilho” 1)	Fase 3, duplo-cego, randomizado; 364 pacientes.	20 países; adultos com IgAN, UPCR ≥0,8 g/g ou proteinúria ≥1 g/d, TFGe 35–90. Predominantemente brancos; asiáticos sub-representados.	**Budesonida** direcionada 16 mg/d por 9 meses vs. placebo, ambos sobre suporte otimizado; seguimento sem fármaco por 15 meses.	2 anos. Coprimários: UPCR em 9 meses e média temporal de TFGe em 2 anos.	Redução relativa do UPCR em 9 meses ~27% vs. placebo; efeito parcialmente persistente após suspensão.	Benefício de TFGe em 2 anos: diferença aproximada de 5,0 mL/min/ 1,73 m^2^ na média temporal; declínio significativamente mais lento.	Mais edema periférico, hipertensão, acne e alterações cushingoides; efeitos sistêmicos não foram abolidos.	FDA: aprovação plena para reduzir a perda de função renal em adultos em risco (Tarpeyo); aprovado também em outras jurisdições.	Primeira terapia específica com demonstração de efeito sobre TFGe após curso finito. Limitações: exclusão de TFGe <35, baixa representação asiática e recorrência de proteinúria após suspensão.
PROTECT (2023; 2 anos) (Rovin et al.)^7^	Duplo antagonismo endotelina A/ angiotensina II	Fase 3, duplo-cego, ativo-controlado; 404 pacientes.	18 países; IgAN, proteinúria ≥1 g/d, TFGe ≥30; população global, majoritariamente branca, com participação asiática relevante.	**Esparsentana** 400 mg 1x/d vs. irbesartana 300 mg 1x/d; titulação inicial 200 vs 150 mg.	Semana 36: UPCR. Até 110 semanas: inclinação de TFGe.	Semana 36: -49,8% vs.-15,1%; redução relativa entre grupos de 41%. Sustentada até 2 anos.	Inclinação crônica favoreceu a esparsentana por ~1,1 mL/ min/1,73 m^2^/ano; inclinação total não atingiu significância no plano original (*p* = 0,058).	Hipotensão, edema e alterações hepáticas; necessidade de monitorização e prevenção de gravidez. Sem sinal de insuficiência cardíaca desproporcional no estudo.	FDA: aprovação plena (Filspari) para retardar a perda de função renal em adultos com IgAN em risco.	Antiproteinúrico muito potente e opção não imunossupressora. Não deve ser associado a IECA/BRA. Benefício de TFGe é favorável, mas o *endpoint* total original ficou limítrofe.
ALIGN – Fase 3 (2026) (Heerspink et al.)^8^	Antagonista seletivo do receptor ETA	Fase 3; duplo-cego; controlado por placebo; *N* = 404 (340 na coorte principal + 64 em estrato com SGLT2i).	24 países; IgAN, proteinúria ≥1 g/d, TFGe ≥30, sobre SRAA máximo. População global; composição racial detalhada deve ser consultada no suplemento.	**Atrasentana** 0,75 mg/d vs. placebo, adicionada ao tratamento de suporte.	Seguimento: 132 semanas (+4 semanas *off-treatment*; análise em 136 semanas).	Proteinúria: redução sustentada (~38% vs. placebo).	TFGe: desaceleração da perda funcional; diferença na inclinação da TFGe de +1,4 mL/ min/1,73 m^2^/ano. TFGe às 132 semanas: diferença de +2,6 mL/min/ 1,73 m^2^	Excelente tolerabilidade; retenção hídrica discreta, sem novos sinais de segurança.	FDA: em processo de conversão da aprovação acelerada para a aprovação tradicional.	Confirma o benefício renal de longo prazo e consolida a atrasentana como terapia antiproteinúrica não imunossupressora direcionada ao eixo da endotelina.
ENVISION (2024) (Mathur et al.)^21^	Anti-APRIL monoclonal (“Gatilho” 1)	Fase 2, duplo-cego, randomizado; 155 pacientes.	Internacional; adultos com IgAN de alto risco sobre suporte. População racialmente diversa, porém o estudo não foi dimensionado para subgrupos.	**Sibeprenlimabe** SC a cada 4 semanas: 2, 4 ou 8 mg/kg vs. placebo por 12 meses.	12 meses. Primário: mudança no UPCR.	Redução do UPCR em 12 meses: ~47,2%, 58,8% e 62,0% nas doses 2, 4 e 8 mg/kg vs. ~20% com placebo; relação dose-resposta.	TFGe permaneceu mais estável nos grupos ativos, mas a fase 2 não foi dimensionada para desfechos renais.	Eventos adversos globalmente semelhantes; infecções respiratórias e reações no local de injeção. Redução de IgA, sem depleção ampla de IgG.	Sem aprovação baseada apenas no ENVISION.	Validou APRIL como alvo causal e apoiou fase 3. Amostra pequena e duração curta.
VISIONARY – análise interina (2025) (Perkovic et al.)^22^	Anti-APRIL monoclonal (“Gatilho” 1)	Fase 3, multicêntrico, duplo-cego; aproximadamente 510 randomizados; análise interina de proteinúria.	Adultos com IgAN confirmada por biópsia e risco de progressão; estudo global. Distribuição racial completa no suplemento.	**Sibeprenlimabe** 400 mg SC a cada 4 semanas vs. placebo por 100 semanas.	9 meses: UPCR; seguimento em curso para TFGe em 24 meses.	Redução relativa ajustada do UPCR vs. placebo de aproximadamente 51,2% em 9 meses.	Desfecho confirmatório de TFGe ainda em andamento na análise interina.	Perfil semelhante ao placebo; infecções e reações de injeção predominantemente leves/ moderadas.	FDA: aprovação acelerada em nov/2025 (Voyxact) para reduzir proteinúria; O benefício renal em longo prazo foi demonstrado na análise final de 104 semanas do estudo VISIONARY, publicada no NEJM em 22/10/2026, evidenciando preservação sustentada da função renal.	Primeiro anti-APRIL aprovado. Forte efeito antiproteinúrico e racional de modificação da doença; benefício funcional de longo prazo ainda aguardado.
ORIGIN-3 – análise interina (2025) (Lafayette et al.)^24^	TACI-Fc: bloqueio dual BAFF/APRIL (“Gatilho” 1)	Fase 3, duplo-cego, controlado por placebo; 431 pacientes.	Global; IgAN, proteinúria persistente e risco de progressão sobre suporte otimizado. Composição racial completa no artigo/ suplemento.	**Atacicepte** 150 mg SC 1x/semana, autoadministrado, vs. placebo.	Semana 36: mudança no UPCR; estudo continua para TFGe.	Diferença relativa ajustada vs. placebo de 41,8%; queda desde o basal ~46% com atacicepte.	Dados definitivos de TFGe ainda pendentes na análise interina publicada.	Reduções de IgA, IgG e IgM; infecções globalmente semelhantes, mas exige vigilância de imunoglobulinas e vacinação.	FDA: aprovação acelerada em 07/07/2026 (Trutakna) para redução de proteinúria; na análise final de 104 semanas do estudo ORIGIN 3, publicada no NEJM em 22/10/2026, evidenciando estabilização do declínio da TFGe	Primeiro bloqueador dual BAFF/APRIL aprovado. Efeito robusto sobre biomarcadores e proteinúria; custo, imunoglobulinas e ausência de desfecho renal final são limitações atuais.
TELIGAN – análise interina (2026) (Lv et al.)^25^	TACI-Fc: bloqueio dual BAFF/APRIL (“Gatilho” 1)	Fase 3, duplo-cego, controlado por placebo; estudo realizado predominantemente na China.	Adultos chineses com IgAN de alto risco; generalização para outras etnias é limitada.	**Telitacicepte** 240 mg SC 1x/semana vs. placebo, além de suporte.	39 semanas: mudança no UPCR; seguimento de função renal em curso.	Redução significativamente maior do UPCR que placebo em 39 semanas; magnitude publicada deve ser apresentada conforme versão final do artigo (aprox. 40–50% de efeito relativo).	Sem comprovação definitiva de preservação de TFGe na análise interina.	Infecções e redução de imunoglobulinas requerem monitorização; perfil geral aceitável na análise interina.	Aprovado na China para algumas doenças autoimunes; situação específica para IgAN varia por jurisdição; não aprovado pela FDA para IgAN até 11/07/2026.	Amplia evidência da estratégia BAFF/APRIL, mas população quase exclusivamente chinesa e ausência de desfecho renal final limitam a extrapolação.
APPLAUSE-IgAN – interina e 24 meses (2025–2026) (Barratt et al.)^19^	Inibidor oral do fator B – via alternativa do complemento (“Gatilho”4)	Fase 3, duplo-cego, controlado por placebo; 518 pacientes na população principal; coorte separada com lesões crônicas graves.	Internacional; IgAN, proteinúria ≥1 g/d, TFGe ≥30 mL/min/1,73 m2 na população principal. População global, com participação asiática e branca.	**Iptacopana** 200 mg VO 2x/d vs. placebo, sobre suporte otimizado.	9 meses: UPCR; 24 meses: inclinação total de TFGe e segurança.	Mês 9: redução relativa ajustada do UPCR de 38,3% vs. placebo.	Dados finais de 24 meses publicados em mar/2026: declínio de TFGe significativamente mais lento com iptacopana; diferença de inclinação favoreceu o tratamento (consultar artigo final para estimativa e IC exatos na versão submetida).	Infecções geralmente semelhantes; risco de infecção por bactérias encapsuladas exige vacinação e educação. Não houve sinal importante de hemólise.	FDA: aprovação acelerada para redução de proteinúria em adultos com IgAN em risco (Fabhalta); dados de 24 meses fornecem evidência confirmatória renal.	Primeira terapia de complemento com evidência de fase 3 de redução de proteinúria e preservação da TFGe. Custo, vacinação e seleção por biomarcadores permanecem questões práticas.

Abreviaturas – TFGe, taxa de filtração glomerular estimada; UPCR, relação proteína/creatinina urinária; SRAA, sistema renina-angiotensina-aldosterona; APRIL, *a proliferation-inducing ligand*; BAFF, *B-cell activating factor*; TACI, *transmembrane activator and CAML interactor*.

Notas – ensaios pivotais de imunossupressão sistêmica e terapias dirigidas à produção de IgA patogênica, endotelina e complemento. Os valores são apresentados conforme a publicação primária.

Resultados de análises interinas de fase 3 não devem ser interpretados como demonstração definitiva de preservação da função renal até a disponibilidade do desfecho de TFGe. Nota crítica: aprovações aceleradas baseadas em redução da proteinúria permanecem condicionadas à confirmação do benefício clínico sobre a função renal.

### Estratégias de tratamento

#### Tratamento renoprotetor de suporte

Nos últimos anos, tornou-se evidente que parte importante da progressão da NIgA decorre da perpetuação de mecanismos intrarrenais de lesão, independentemente da persistência do estímulo imunológico inicial. A ativação de vias relacionadas à hipertensão intraglomerular, à disfunção podocitária, à inflamação túbulo-intersticial e à fibrose progressiva passou a ser reconhecida como um importante alvo terapêutico.

Dessa forma, é amplamente aceito atualmente que pacientes com NIgA devem receber cuidados renoprotetores de suporte individualizados e otimizados. Essa prescrição pode anteceder ou ser iniciada concomitantemente com a terapia “imunossupressora”, conforme o caso para alguns, ou conforme uma abordagem terapêutica predefinida para outros^4^, aspecto esse que ainda é motivo de discussão.

Os cuidados de suporte incluem modificações no estilo de vida, como dieta saudável, exercícios físicos, controle de peso, cessação do tabagismo, restrição de sódio, controle da pressão arterial sistêmica e medidas para redução do risco cardiovascular. Entre os recursos medicamentosos disponíveis, citam-se os tratamentos com: (1) inibidores do sistema renina-angiotensina (SRAA); (2) inibidores do SGLT2; (3) antagonistas do receptor de mineralocorticoide e (4) antagonistas do receptor de endotelina A. Alguns desses tratamentos tiveram seu papel definido na doença renal crônica e em glomerulopatias proteinúricas em geral, como o bloqueio do SRAA e os inibidores do SGLT2, e também na NIgA^5,6^. Esses fármacos já foram incorporados ao tratamento rotineiro globalmente.

Ainda no contexto da proteção renal, os antagonistas do receptor da endotelina representam uma das estratégias mais promissoras. A endotelina-1 exerce papel relevante na lesão podocitária, no aumento da permeabilidade glomerular, na vasoconstrição intrarrenal e na progressão da fibrose. O estudo PROTECT demonstrou que a esparsentana, antagonista dual dos receptores ETA e AT1, promoveu redução sustentada da proteinúria, acompanhada de desaceleração do declínio da função renal, em comparação à irbesartana, consolidando-se como uma importante alternativa para pacientes com NIgA em risco de progressão^7^. Mais recentemente, o estudo ALIGN confirmou que a inibição seletiva do receptor ETA com atrasentana também reduziu significativamente a proteinúria quando adicionada ao tratamento de suporte otimizado, reforçando o papel da via da endotelina como alvo terapêutico independente da modulação imunológica^8^.

Outra estratégia que ganhou relevância foi o antagonismo não esteroidal do receptor de mineralocorticoide. Embora inicialmente desenvolvido para doença renal diabética, o estudo FIND-CKD demonstrou que a finerenona também foi capaz de reduzir albuminúria e proteinúria e retardar o declínio da TFG estimada (TFGe) em pacientes com doença renal crônica não diabética, incluindo uma ampla população com glomerulopatias e um subgrupo expressivo de pacientes com NIgA^9^.

A seguir, são brevemente descritas outras estratégias terapêuticas, incluindo alguns tratamentos “imunossupressores” já adotados no manejo da NIgA, bem como ensaios clínicos que ilustram a evolução do tratamento dessa glomerulopatia ao longo dos anos.

#### Corticosteroides

Embora estudos mais antigos descrevessem benefícios reais com o uso de corticosteroides sistêmicos na NIgA, a corticoterapia tem sido alvo de críticas e preocupações nos últimos anos, o que, em grande parte, se deve a estudos clínicos mais recentes que documentaram as fragilidades e os eventos adversos dessa terapia.

É preciso destacar, entretanto, que, em algumas condições clínicas, o uso de corticosteroide sistêmico na NIgA continua inquestionável, como na glomerulonefrite rapidamente progressiva e na síndrome nefrótica^10^, até que alternativas realmente eficientes sejam testadas.

O estudo STOP-IgAN (*Intensive Supportive Care plus Immunosuppression in IgAN*) representou um importante ponto de inflexão na história do tratamento da NIgA. Após um período de otimização rigorosa do tratamento de suporte, a adição de imunossupressão sistêmica (corticosteroides para todos, com adição de ciclofosfamida seguida de azatioprina se a TFGe estivesse entre 30 e 59 mL/min/1,73 m^2^) não resultou em benefício sustentado na preservação da função renal em longo prazo, apesar da maior frequência de remissão clínica e redução da proteinúria em parte dos pacientes. Além disso, o aumento de eventos adversos reforçou a necessidade de uma seleção mais criteriosa dos candidatos à imunossupressão sistêmica^11,12^.

Alguns anos mais tarde, o estudo TESTING trouxe uma nova perspectiva ao demonstrar que a metilprednisolona foi capaz de reduzir significativamente o risco de progressão renal em pacientes cuidadosamente selecionados. No entanto, a interrupção precoce da primeira fase do estudo (TESTING-1, *Effect of Oral Methylprednisolone on Decline in Kidney Function or Kidney Failure in Patients with IgA Nephropathy Randomized Clinical Trial*), que avaliou a dose de 0,8 mg/kg/dia de metilprednisolona, devido ao aumento de infecções graves, evidenciou que a eficácia dos corticosteroides precisava ser equilibrada com a sua toxicidade. A estratégia posteriormente adotada (TESTING-2), utilizando doses menores (metilprednisolona 0,4 mg/ kg/dia, VO) associadas à profilaxia para *Pneumocystis jirovecii*, manteve benefício clínico relevante com melhor perfil de segurança, consolidando o papel dos corticosteroides como opção terapêutica para pacientes de alto risco, porém longe de representar uma estratégia universal para todos os indivíduos com NIgA^13^.

Uma oportunidade de uso de corticosteroides na NIgA com menos eventos adversos surgiu com a utilização bem-sucedida da budesonida de liberação entérica, que representou uma mudança conceitual importante no tratamento da NIgA. Essa estratégia foi desenvolvida para atuar preferencialmente sobre o tecido linfoide associado ao intestino distal, considerado um dos principais locais de produção da Gd-IgA1. Dessa forma, procurou-se reduzir a atividade imunológica relacionada à doença, evitando-se a exposição sistêmica aos corticosteroides.

Os estudos NEFIGAN e, posteriormente, NefIgArd demonstraram redução significativa e sustentada da proteinúria, acompanhada pela desaceleração do declínio da função renal em pacientes com NIgA e risco elevado de progressão, estabelecendo a budesonida direcionada como a primeira terapia especificamente aprovada para essa doença^14,15^. Além da eficácia clínica, esses estudos reforçaram um conceito que se tornaria central no desenvolvimento terapêutico da NIgA: a possibilidade de interferir seletivamente em etapas específicas da fisiopatologia da doença, em vez de promover imunossupressão sistêmica inespecífica.

#### Ciclofosfamida, micofenolato e outros imunomoduladores

Vale lembrar que, além dos corticosteroides, a ciclofosfamida e a azatioprina, geralmente em associação com os primeiros, surgiram como opções para casos de NIgA com apresentação proteinúrica. Além disso, o micofenolato de mofetila (MMF) é citado em diretrizes internacionais^10^ como uma alternativa, mas a recomendação é restrita apenas a pacientes chineses, nos quais o MMF demonstrou preservar a função renal e permitir a redução da exposição a corticosteroides em ensaios clínicos randomizados com acompanhamento de longo prazo, o que até o momento não se confirmou em pacientes caucasianos^16^. Também é descrito benefício da hidroxicloroquina quando utilizada em pacientes chineses, mas não em outras populações^17,18^.

#### Inibidores do complemento

Tem-se demonstrado que a ativação das vias alternativa e das lectinas exerce papel central na progressão da NIgA. A presença frequente de depósitos glomerulares de C3, properdina, C4d e outros componentes do complemento associa-se a uma maior atividade da doença e a um pior prognóstico, fornecendo o racional para o desenvolvimento de moléculas capazes de modular seletivamente essas vias.

Entre essas estratégias, a inibição do fator B da via alternativa tornou-se aquela com maior evidência clínica até o momento. O estudo APPLAUSE-IgAN demonstrou que a iptacopana, um inibidor oral seletivo do fator B, promoveu redução significativa e sustentada da proteinúria, acompanhada por desaceleração do declínio da TFG e pela redução do risco de desfechos renais compostos durante o seguimento. Esses resultados consolidaram a via alternativa do complemento como um alvo terapêutico relevante na NIgA e levaram à aprovação regulatória da iptacopana para pacientes com risco de progressão^19^.

Além desse, outros fármacos que inibem diferentes pontos na cascata do complemento estão sob investigação e há indícios de que reduzem a proteinúria. Os alvos a que se dirigem são: C3 (ex.: pegcetacoplana), C5 (ex.: ravulizumab), receptor de C5a, fator D da via alternativa e MASP-2 da via das lectinas, entre outros^20^.

#### Fármacos direcionados à sinalização BAFF e/ou APRIL

As primeiras terapias sistêmicas capazes de interferir diretamente na produção da Gd-IgA1, principal evento desencadeador da doença, tiveram como objetivo atuar precocemente na cascata fisiopatológica, reduzindo a geração dos imunocomplexos danosos aos rins antes mesmo da deposição glomerular. Entre os alvos estudados, a modulação das citocinas APRIL (*A Proliferation-Inducing Ligand*) e BAFF (*B-cell Activating Factor*) representa, até o momento, a abordagem mais avançada.

APRIL e BAFF exercem papel central na sobrevivência, diferenciação e maturação das células B e plasmócitos, regulando a produção de imunoglobulinas, particularmente IgA. Concentrações aumentadas dessas citocinas em pacientes com NIgA correlacionam-se com níveis mais elevados de Gd-IgA1, maior proteinúria e maior risco de progressão da doença.

A primeira estratégia consistiu no bloqueio seletivo de APRIL. O estudo ENVISION demonstrou que o sibeprenlimabe promoveu redução significativa da proteinúria, acompanhada por uma redução sustentada da IgA sérica e da Gd-IgA1, confirmando que a inibição seletiva de APRIL poderia modificar biomarcadores diretamente relacionados à fisiopatologia da doença. Posteriormente, os resultados do estudo de fase 3 VISIONARY confirmaram esses achados em uma população maior, consolidando o sibeprenlimabe como a primeira terapia anti-APRIL aprovada para pacientes com NIgA em risco de progressão^21,22^.

Paralelamente, outra estratégia buscou ampliar essa modulação imunológica por meio do bloqueio simultâneo de APRIL e BAFF. O atacicepte, proteína de fusão derivada do receptor TACI, determinou reduções consistentes da proteinúria e dos níveis circulantes de IgA e Gd-IgA1 no estudo ORIGIN, resultados posteriormente confirmados na fase 3 do ORIGIN-3^23,24^. Em julho de 2026, o atacicepte tornou-se o primeiro agente com bloqueio combinado de APRIL e BAFF aprovado pela FDA para redução da proteinúria em pacientes com NIgA. O telitacicepte, que compartilha mecanismo semelhante, também determinou reduções expressivas da proteinúria em estudos de fase 3, reforçando o potencial terapêutico dessa alternativa, embora ainda sejam necessários dados adicionais sobre preservação da função renal e segurança em longo prazo^25^.

Deve-se esclarecer que comparações diretas entre os diversos inibidores de APRIL e BAFF não são apropriadas, uma vez que os ensaios clínicos diferiram quanto aos critérios de inclusão, tratamento de suporte, duração do seguimento e desfechos avaliados. Até o momento, não existem evidências que permitam afirmar superioridade de uma molécula sobre a outra. Além disso, mais importante do que a comparação entre medicamentos é o conceito introduzido por essa classe terapêutica. Pela primeira vez, tornou-se possível interferir sistemicamente na produção da IgA patogênica por meio de mecanismos seletivos, aproximando o tratamento da NIgA de uma estratégia baseada nos mecanismos da doença, e não apenas na supressão inespecífica da resposta imune.

#### Anti-CD38 e outros

Também estão em investigação fármacos anti-CD38, que objetivam depletar os plasmócitos CD38(+), os quais seriam responsáveis pela produção de Gd-IgA1 e de autoanticorpos contra Gd-IgA1, com resultados iniciais promissores^26^. Inibidores da inflamação dos podócitos (anti-suPAR) e outros agentes também estão em investigação^27^.

## CONCLUSÃO

Houve certamente muitas mudanças nos últimos anos em relação à oferta de opções terapêuticas para a NIgA, assim como para outras doenças glomerulares, após décadas sem investimento nessa área.

Uma melhor compreensão dos mecanismos de desenvolvimento da doença contribuiu para o desenvolvimento de medicamentos direcionados a alvos específicos nas vias patogênicas. Tais tratamentos podem oferecer uma nova maneira de retardar ou, eventualmente, interromper os danos renais na NIgA.

Se, por um lado, é alentador dispor hoje de várias opções de tratamento, por outro, é desafiador escolher a melhor estratégia. A título de exemplo, podemos discutir o que é possível fazer hoje em um país como o Brasil. Antes de mais nada, é preciso lembrar que o tratamento vai depender da apresentação da NIgA, no que tange às suas características clínico-laboratoriais e histopatológicas, assim como das peculiaridades do paciente, como comorbidades, características genéticas, entre outras. Contamos com quase todos os recursos renoprotetores aqui citados. Podemos e devemos usá-los em combinação, buscando a menor proteinúria e a preservação da TFG. Assim que disponíveis, os fármacos anti-endotelina serão certamente uma alternativa útil a ser utilizada em combinação com o bloqueio do SRAA, em especial, e também com os demais. Efeitos adversos deverão ser ponderados na seleção dessas combinações, sabendo-se que, até o momento, considera-se o bloqueio do SRAA um recurso inicial ao qual se somam, conforme necessário, algumas das outras alternativas. A adição de imunossupressão também dependerá da apresentação, buscando-se identificar qual o paciente com maior potencial de benefício. Até o momento, a corticoterapia sistêmica é a base do tratamento nos casos de síndrome nefrótica e glomerulonefrite rapidamente progressiva, com a possibilidade de associar outros imunossupressores. A maior dificuldade é lidar com a miríade de apresentações não nefróticas com e sem déficit de TFG, em que é preciso definir o quanto é pertinente investir na manipulação do sistema imune e por quanto tempo, sabendo-se que o que está disponível na rotina ainda são os corticosteroides, com recomendação de uso de doses menores nessas situações. Vale destacar que, nos dias atuais, faltam marcadores laboratoriais e/ou histopatológicos que definam com clareza o que fazer, ou mesmo um instrumento que defina categorias de risco para a NIgA e contribua para o processo de decisão^28^. Persistência de proteinúria, apesar do tratamento renoprotetor, hematúria mais expressiva e pouca cronicidade na biópsia renal são indicadores gerais de que existe atividade com potencial de resposta e, quando disponíveis e aprovadas para esse fim, opções de modulação imune podem ser utilizadas, pesando-se a relação custo-benefício caso a caso. É notório que, diante da preocupação com o potencial de progressão, tem-se estimulado inclusive o encaminhamento de pacientes com NIgA proteinúrica a procurarem opções de tratamento ainda em investigação em centros que desenvolvem pesquisa clínica^10^.

Parte dos novos medicamentos aqui discutidos já foi aprovada para NIgA, mas, ainda que disponíveis comercialmente, seus custos são elevados, limitando o acesso dos pacientes a eles, o que é mais um elemento a ponderar na hora de prescrevê-los. Assim, deve-se manter um olhar crítico e aguardar que sejam definidas com clareza suas indicações nas diferentes apresentações e fases de evolução da NIgA.

Por fim, como já esclarecido, citamos neste artigo apenas alguns fármacos para dar uma ideia do contexto em que se encontram os recursos terapêuticos destinados à NIgA. Neste momento, mais estudos são necessários para definir qual a combinação mais adequada de tratamentos. Porém, já se pode dizer que não será uma prescrição única para uma “doença única”, e sim diferentes combinações para os vários endótipos dessa glomerulopatia, ajustadas de forma personalizada à doença de cada paciente e às suas demandas, incluindo seu bem-estar e qualidade de vida.

## Data Availability

Nenhum dado novo foi gerado ou analisado neste estudo.
